# SLEEP MEASUREMENT TECHNOLOGY IN THE 40 WINKS PRAGMATIC CLINICAL TRIAL: CHALLENGES AND SOLUTIONS

**DOI:** 10.1093/geroni/igae098.1371

**Published:** 2024-12-31

**Authors:** Kathy Richards, Brian Cox, Vanessa Aguilar, Regina George, Kate Smith, Sophia Geisser, Liam Fry, Robert Morgan

**Affiliations:** The University of Texas Austin, The Woodlands, Texas, United States; Alabama Research Institute on Aging, Tuscaloosa, Alabama, United States; University of Alabama, Tuscaloosa, Alabama, United States; University of Alabama, Tuscaloosa, Alabama, United States; University of Alabama, Tuscaloosa, Alabama, United States; University of Alabama, Tuscaloosa, Alabama, United States; The University of Texas Austin, Austin, Texas, United States; The University of Texas Health Science Center, Houston, Texas, United States

## Abstract

This presentation describes examples, challenges, and solutions for using technology as part of a pragmatic trial to both deliver a sleep promotion intervention and measure its outcomes. In this trial, target nursing home residents wear an actigraph on their wrist for one week. Actigraphs are a non-invasive way of monitoring activity and rest cycles. One effective and novel application of this technology is use of the actigraphy clinical report. As part of the intervention, the clinical report of the resident’s nighttime sleep, daytime napping, light exposure, and physical activity is shared with the nursing home staff during frontline staff huddles. The frontline staff then use the report to inform and target their sleep promotion interventions, for example, increasing daytime light exposure or activity, and to monitor effectiveness. Use of this technology is not without challenges. We will thus discuss several challenges and their solutions, including identifying the best wrist bands for securing the actigraphs and addressing manufacturer discontinuation of actigraph production and hardware service. We will also describe the trial’s initial pilot that compared the gold standard research actigraph with a less expensive and more readily available consumer-focused device and why we, ultimately, chose to use only the research device.

